# Anthropic Pressure on Cetaceans Stranded Along the Ligurian Coast Within the Pelagos Sanctuary: A Case Series

**DOI:** 10.3390/ani14223207

**Published:** 2024-11-08

**Authors:** Virginia Mattioda, Federica Giorda, Guia Consales, Camilla Testori, Simona Zoppi, Maria Goria, Maria Ines Crescio, Laura Serracca, Katia Varello, Valerio Carta, Letizia Marsili, Matteo Baini, Matteo Galli, Cristina Cristina Fossi, Elena Fontanesi, Fulvio Garibaldi, Guido Pietroluongo, Sandro Mazzariol, Francesco Brunelli, Cristina Casalone, Carla Grattarola

**Affiliations:** 1 Istituto Zooprofilattico Sperimentale del Piemonte, Liguria e Valle d’Aosta, 10154 Torino, Italy; virginia.mattioda@izsplv.it (V.M.); camilla.testori@izsplv.it (C.T.); simona.zoppi@izsplv.it (S.Z.); maria.goria@izsplv.it (M.G.); mariaines.crescio@izsplv.it (M.I.C.); laura.serracca@izsplv.it (L.S.); katia.varello@gmail.com (K.V.); valerio.carta@izsplv.it (V.C.); cristina.casalone@izsplv.it (C.C.); carla.grattarola@izsplv.it (C.G.); 2 Department of Physical, Earth and Environmental Sciences, University of Siena, Via Mattioli, 4, 53100 Siena, Italy; letizia.marsili@unisi.it (L.M.); matteo.baini@unisi.it (M.B.); matteo.galli@unisi.it (M.G.); fossi@unisi.it (C.C.F.); 3 Centro Interuniversitario di Ricerca Sui Cetacei (CIRCE), University of Siena, 53100 Siena, Italy; 4 Delfini del Ponente APS, Via Regione Bussi 27, 18100 Imperia, Italy; elena.fontanesi2@gmail.com; 5 DISTAV, University of Genoa, Corso Europa 26, 16132 Genova, Italy; fulvio.garibaldi@unige.it; 6 Department of Comparative Biomedicine and Food Science, University of Padua, 35020 Legnaro, Italy; guido.pietroluongo@gmail.com (G.P.); sandro.mazzariol@unipd.it (S.M.); 7 AST Ancona, 261100 Ancona, Italy; francesco.brunelli@sanita.marche.it

**Keywords:** dolphins, Ligurian sea, Pelagos Sanctuary, anthropic pressure, striped dolphin, common bottlenose dolphin, fishery interaction, organochlorine compounds, terrestrial pathogens, marine litter

## Abstract

Data collected by C. Re. Di. Ma over a 3-year period (2020–2022) were considered to assess anthropic pressure on cetaceans living in the Ligurian sea. Out of a total of 37 stranded cetaceans, a complete *post mortem* examination was performed on 23 cases. Of these, 14 were further selected considering at least one of these conditions: (i) confirmed, probable, or suspected interaction with fishing activities through the application of a standardized diagnostic framework (7/14; 50%), (ii) toxicological stress through the evaluation of OCs hazardous levels (14/14; 100%), and (iii) terrestrial pathogen-associated disease (systemic infection and/or associated lesions) (7/14; 50%). For 9 animals out of a total of 14 selected, the cause of death was classified as natural (6/14; 42,8%), anthropic (3/14; 21,4%), or not determined (5/14; 35,7%) based on gross and histological pathology and ancillary testing. These findings extend our knowledge of the anthropic pressure to which cetaceans stranded along the Ligurian coastline are subjected from a multidisciplinary point of view.

## Simple Summary

In recent decades, environmental changes consequent to human activities have become more important factors in the conservation of wild species. Despite the Ligurian sea being part of the Pelagos Sanctuary, which has been a Specially Protected Areas of Mediterranean Importance (SPAMI) and a Marine Protected Area (MPA) since 1999, several anthropogenic threats affecting the health of cetacean populations living in this area are known to be present. In particular, interaction with fishing activities, contamination with chemical compounds such as organochlorine compounds (OCs), interaction with marine debris (especially, plastic debris), and the emergence of infectious agents, many of which are of terrestrial origin as a result of contamination of the marine environment by agricultural, animal, and human waste, are just some of the threats. In this regard, cetaceans stranded along the Ligurian coast between 2020 and 2022 were evaluated by means of a comprehensive *post mortem* examination and thorough specific diagnostic investigations to assess the level of anthropic pressure in this area by the Italian National Reference Center for Diagnosis of stranded Marine Mammals (C. Re. Di. Ma).

## 1. Introduction

The Pelagos Sanctuary represents the first international marine area in the Mediterranean Sea dedicated to the protection of marine mammals and their habitats. This Specially Protected Area of Mediterranean Importance (SPAMI) and Marine Protected Area (MPA) was established in 1999 under an international agreement between France, Italy, and the Principality of Monaco. This area covers 87,500 km^2^ and 2022 km of coast between south-eastern France, Monaco, north-western Italy and northern Sardinia, surrounding Corsica and the Tuscan Archipelago [1]. The Sanctuary was established mainly for three reasons: the discovery of a high number of various cetacean species in the Ligurian Sea; the emergence of remarkable threats to these species following a striped dolphin (*Stenella coeruleoalba*) epizootic in 1990, and the lack of an adequate legal framework for the protection of this highly cetacean-populated area [1]. 

During the 1990s, Liguria was the most polluted region in Italian waters due to persistent organic pollutants (POPs) [2], and today, the coasts of the Ligurian Sea remain among the most urbanized and industrialized along Italy’s coastline [3]. Over the past few decades, there has been growing concern about the effects of environmental stress, particularly human activities, on marine mammals [4,5]. Human-driven environmental changes are increasingly recognized as significant factors in the conservation of wild species [6,7]. Along the Ligurian coastline, several anthropogenic threats impact cetacean populations. These include interactions with fishing activities [8,9,10], contamination from chemicals like organochlorines (OCs) [11,12,13,14], marine debris (especially plastic) [15,16,17,18,19,20,21], and the spread of infectious agents, many originating from terrestrial sources due to the contamination of the sea by agricultural, animal, and human waste [7,14,22,23,24,25,26,27,28,29,30,31,32,33,34,35,36].To assess the impact of these threats on cetacean health, it is crucial to systematically investigate stranding events through comprehensive *post mortem* examinations and detailed diagnostic investigations. Such events provide valuable insights into the health of the population, allowing not only an assessment of mortality rates but also an evaluation of the risks posed by human activities [37].

Since the causes of strandings are generally multifactorial, it is necessary to put together several factors that may act synergistically; for example, nutritional stress due to reduced prey availability and toxicological stress due to anthropogenic chemical contaminants, such as persistent organic pollutants (POPs), may act together when malnutrition promotes the mobilization of these lipophilic contaminants from the blubber, thus making the animals more exposed to their toxic effects when they are already debilitated by inadequate nutrition [8,14]. Moreover, these chemical pollutants act as endocrine disruptors, reducing their reproductive success and depressing their immune system, also making cetaceans more susceptible to infectious diseases [7,12,13,14]. 

The present study aims at monitoring various levels of human activities as a direct and indirect threat to cetaceans. In particular, animals stranded along the Ligurian coast between 2020 and 2022 that had undergone a complete *post mortem* examination were selected according to the presence of specific factors associated with anthropic pressure, such as fishery interaction, toxicological stress by OCs compounds, and systemic infection and/or associated lesions caused by pathogens of terrestrial origin. Furthermore, marine debris ingestion was assessed through a standardized method, in order to confirm the presence of macro-, meso- and microlitter in the gastrointestinal tracts (GIT) of stranded cetaceans.

## 2. Materials and Methods

### 2.1. Post Mortem Examination

As the National Reference Center for Diagnosis of stranded Marine Mammals (C. Re. Di. Ma.), at the Diagnostic Laboratory of Imperia of the Istituto Zooprofilattico Sperimentale (IZS), we advance research on marine mammals through the examination of stranded animals along the Italian coastline [38,39,40,41,42,43,44,45].

Among the total of 37 cetaceans stranded dead in the study area between 2020 and 2022, thanks to the activities carried out by C. Re. Di. Ma., interventions were possible on 30 animals. A complete *post mortem* examination, according to internationally standardized guidelines [46], was conducted on 23 individuals [43,44,45].

Of these, 14 cases were further selected according to the presence of at least one of the three following conditions, considered to be of anthropogenic origin, regardless of whether or not there was a connection with the hypothesis of cause of death: (i) confirmed, probable or suspected interaction with fishing activities, assessed with a standardized diagnostic framework [47]; (ii) toxicological stress relief, assessed with the evaluation of organochlorine compounds (OCs), hazardous levels of hexachlorobenzene (HCB), polychlorinated biphenyls (PCBs), and dichlorodiphenyltrichloroethane (DDTs) [12,48]; and (iii) terrestrial pathogen-associated disease, assessed with diagnostic evaluation of systemic infection and/or associated lesions. 

**Figure 1:**
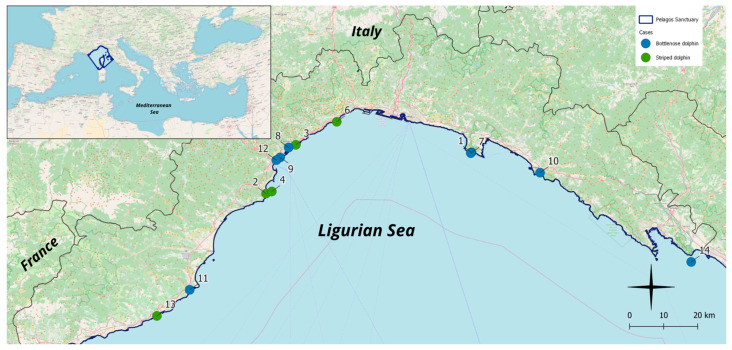
Map of the study area (Ligurian coastline), displaying the stranding locations of the 14 cases selected for the study (8/14 = bottlenose dolphin and 6/14 = striped dolphin). Upper inset: Mediterranean Sea and Pelagos Sanctuary perimeters (QGIS 3.22.5. QGIS Geographic Information System. Open Source Geospatial Foundation Project. http://qgis.osgeo.org, accessed on 16 October 2024).

Each case was assigned both an IZS identification code and a code from the Banca Dati Spiaggiamenti (BDS) (http://mammiferimarini.unipv.it, accessed on 23 April 2024). Then, all cases were coded in chronological order, from Case 1 to Case 14 (Figure 1). 

During *post mortem* examination of each case, general data collection regarding stranding date and location, sex, total body length (TBL) in centimeters, weight in kilograms, estimated age class, sexual maturity, decomposition condition category (DCC), nutritional condition category (NCC), gastric contents, and macroscopic lesions and parasites, was performed. All these parameters are part of the routine *post mortem* data collection and allow us to assess the carcass conditions completely.

Normally, a full investigation with subsequent and additional examinations is indeed highly recommended, but if this cannot be carried out for any reason, it is always good practice to collect at least the following data: species, sex, stranding location, stranding date, and total body length to estimate age class [46]. 

Starting from the TBL, which corresponds to the length of the carcass in centimeters from the anterior end of the head to the interlobar sinus of the caudal fin, it was possible to define the estimated age class also considering the maturation of the gonads. The estimated age class was reported as newborns/calves, juveniles, or adults [49,50]. For cases with amputated tails, the TBL was estimated considering the fact that the length of the tail represents about 1/9–1/10 of the TBL [14].

The decomposition condition category (DCC) of the carcasses at the time of necropsy was classified as follows: code 1 (extremely fresh carcass, just dead), code 2 (fresh), code 3 (moderate decomposition), code 4 (advanced decomposition), or code 5 (mummified or skeletal remains) [46].

The nutritional condition category (NCC) was determined based on anatomical parameters such as the convexity of the dorsal profile, rib prominence, and the amount of visceral fat and blubber thickness measured immediately anterior to the dorsal fin at three locations (dorsal, lateral, and ventral) [46] and was categorized as good, moderate, or poor.

Considering macroscopic findings, these were photographed and recorded during the examination; in addition, any presence of gas bubbles at the macroscopic level within the main vessels was assessed according to Bernaldo et al., 2013 [51]. Tissue samples from all main organs and lesions were collected into aliquots for subsequent analyses. Specifically, for microbiological and toxicological investigations, aliquots were kept frozen at −20 °C, for biomolecular analyses at −80 °C, and the rest was preserved in 10% buffered formalin for histological and immunohistochemical (IHC) investigations. Blood serum, aqueous humor, pericardial fluid, and cerebrospinal fluid (CSF), when available, were kept frozen at −20 °C for serological investigations.

Regarding the presence of parasites, this was evaluated both through macroscopic and microscopic examination of tissues and, when present, preserved in 70% alcohol for microscopic identification, following established morphological characteristics.

Additionally, a multidisciplinary analysis of the gastrointestinal tract (GIT) was performed following the approach of Corazzola et al., 2021 [52], to assess specific pathological evaluations, microbiological and virological analyses, algal biotoxin detection, diet, parasitological investigations, and the presence of marine litter.

### 2.2. Diagnostic Investigations

Diagnostic analyses were performed from aliquots taken during necropsy examinations according to internationally standardized guidelines [46] and additional sampling was considered depending on gross findings at the necropsy. The number and type of analyses were mainly influenced by the DCC of the individual cases at the time of the necropsy. All diagnostic investigations performed for this study are reported in Table 1.

All samples were collected and fixed in 10% neutral buffered formalin, embedded in paraffin, sectioned at 4 ± 2 µm, stained with hematoxylin and eosin (HE), and examined under a light microscope. For brain examinations, different areas were sampled and analyzed, including the basal nuclei, thalamus, mesencephalon, pons, obex, and the frontal, parietal, occipital, and cerebellar cortex, following Giorda et al. [53].

Immunohistochemistry (IHC) for *Morbillivirus* was systematically performed on brain tissues using a monoclonal anti-Canine distemper virus (CDV) antibody (VMRD, Pullman, WA, USA). IHC for *Toxoplasma gondii* was conducted only on PCR-positive brain tissues or when histological changes suggestive of the infection were present, using a polyclonal serum of caprine origin (VMRD, Pullman, WA, USA) [22].

Bacteriological investigations were carried out on available tissue samples based on the degree of tissue decomposition, along with tissues showing gross evidence of suspected bacterial infection, for standard aerobic, anaerobic, and microaerobic (5% CO_2_) bacterial culture and identification through biochemical and/or molecular analyses.

Additionally, samples from target tissues underwent specific bacteriological procedures to screen for *Listeria* spp., *Salmonella* spp., and *Brucella* spp., according to international recommendations [54].

Molecular detection of *Cetacean morbillivirus* (CeMV) [55], *Herpesvirus* (HV) [56], *T. gondii* [57], *Brucella* spp. [58], and *Photobacterium damselae subsp. damselae* (Pdd) [59] was performed on available target tissues. Molecular detection of *Poxvirus* [60] was also attempted on skin lesions that grossly suggested the infection.

Serological analysis for the detection of *T. gondii*, *Brucella* spp., and *Morbillivirus* antibodies was also conducted [22,61].

### 2.3. Cause of Death Evaluation

The likely cause of death (COD) was hypothesized based on the findings of *post mortem* investigations and ancillary analyses. 

In particular, the hypotheses of the CODs were classified as natural when related to infectious diseases or non-infectious conditions (i.e., senescence, neonatal/perinatal pathologies, neoplasia, etc.) [62,63] and as anthropic when related to an interaction with human activities such as fishery interaction, vessel collision, or marine litter ingestion [47].

Moreover, specific histology and IHC investigations were performed to confirm capture myopathy associated with fishery interaction [64,65].

Starting from 2020, Life DELFI’s framework for fishery interaction [47] was adopted to better assess cetacean *post mortem* findings. According to the framework categories, findings related to fishery interaction were categorized as “by-catch with active fishing gear”, “by-catch with passive fishing gear”, “chronic entanglement”, “larynx entanglement”, “ingestion”, “intentional injury”, and “by-catch with not determined fishing gear” whenever the bycatch event could not be identified with active or passive fishing gear. 

For the assessment of fishery interaction, most evidence requires a DCC of the carcass between 1 and 3; based on this, fishery interaction was categorized as confirmed (C), probable (P), or suspect (S) [47].

### 2.4. Toxicological Analysis 

Toxicological analyses were conducted by the Department of Physical Sciences, Earth and Environment at the University of Siena, following the U.S. Environmental Protection Agency (EPA) 8081/8082 method (https://www.epa.gov/sites/default/files/2015-12/documents/8081b.pdf, accessed on 23 April 2024) with modifications as reported in Grattarola et al., 2023, [14,66] Briefly, organochlorine (OCs) levels were examined in the subcutaneous adipose tissue (blubber) of all cases. Samples (5–20 g) were freeze dried and then extracted with n-hexane using a Soxhlet. Samples were then purified with 95% sulfuric acid and with liquid chromatography on a column containing Florisil. OCs were determined with an Agilent 6890N series gas chromatograph equipped with a 63Ni electron capture detector (ECD) (Agilent, Santa Clara, CA, USA).

To evaluate the potential hazard for PCBs and DDTs in striped dolphins (*Stenella coeruleoalba*), the CAN equation proposed by Marsili et al. 2004 has been applied, expressed as follows:


CAN (Potential hazard) = (9.51 × 10^−6^ × PCBs + 4.40 × 10^−6^ × DDTs) − 0.92

This model is only valid for Mediterranean striped dolphins and was calculated to obtain a different key for interpreting data on OCs accumulation in the blubber of Mediterranean striped dolphins [12].

### 2.5. GIT Analysis and Marine Litter Investigations 

To ensure a multidisciplinary investigation of the GIT of stranded marine mammals, a multi-sieve tool was used according to Corazzola et al. 2021 [52]. 

In detail, organs (stomach and/or intestine) were first sealed with a string at the cranial and caudal levels to minimize the contamination from environmental sources of micro-litter items, then rinsed externally and weighted. Each organ was opened longitudinally to collect its content in a tank and record any gross lesions or collect any parasitic elements. Also, samples for histological, virological, and bacteriological examinations were collected to carry out the analysis. 

The content was then transferred into the first sieve (20 mm mesh) and rinsed with water to make it proceed towards the next sieves. The content was made to proceed through the next sieves, collecting in 4 different containers any marine debris, parasites, or alimentary residues visible to the naked eye present in the 1000 µm, 500 µm, 250 µm, and 100 µm sieves.

Considering marine litter analysis, a standardized protocol to quantify and characterize macro-, meso- and microplastics was defined by the Department of Physical, Earth and Environmental Sciences at the University of Siena, based on the criteria indicated by the European working group for descriptor D10 of the European Marine Strategy Framework Directive (2008/56/EC) [67,68]. 

In detail, litter items retained on sieves with apertures larger than 1 mm were individually isolated, counted, and their dry mass determined to a precision of 0.01 g following desiccation at room temperature for a minimum of 24 h. To isolate particles from sieves with apertures smaller than 1 mm, a 1/20 solution of 10% KOH was employed to digest and remove excess organic matter. 

The samples were then incubated in a thermostatic bath at 50 °C for 18 h, filtered through a 50 µm mesh, and examined using a stereomicroscope equipped with a micrometric eyepiece. All isolated particles were subsequently photographed, measured, and categorized according to their maximum length as macroplastics (>25 mm), mesoplastics (5–25 mm), or microplastics (<5 mm). 

## 3. Results

### 3.1. Post Mortem Examination

Based on the evidence of direct or indirect anthropic pressure, without any consideration of the hypothesis of the cause of death, 14 cases were selected for this study out of 23 individuals for a complete *post mortem* examination. 

In detail, 50% (7/14) of total selected cases showed confirmed, probable, or suspected interaction with fishing activities [47]; 100% (14/14) of total cases showed levels of OCs compounds compatible with toxicological stress [12,48] and 50% (7/14) of total cases reported terrestrial pathogen systemic infections and/or associated disease. 

Considering the presence of marine litter in the GIT, the multidisciplinary investigation of the GIT of stranded animals was performed only in 43% (6/14) of total cases; for this reason, this parameter was not considered as a selection criterion but was nevertheless considered retrospectively for the analyzed cases [52,67,68]. 

Regrettably, the DCC restricted the *post mortem* investigations in certain cases, making it impossible to hypothesize the cause of death (COD). In addition, no confirmed, probable or suspected case of vessel collision was registered during the study period. All data are reported in Table 2. 

Out of the total number of selected cases, 57% (8/14) were recognized as bottlenose dolphins and 43% (6/14) as striped dolphins; among them, the majority were male, with 71.4% (10/14) of total cases, while 21.4% (3/14) were female. For one case (case 6), it was not possible to define the sex because of the absence of the distal part of the carcass. 

Considering the estimated age class, 36% (5/14) of cases were classified as adults, 43% (6/14) as juvenile, and 14.2% (2/14) as newborns/calves. For one case (case 6), it was not possible to estimate the age class due to the absence of the distal part of the carcass. 

The DCC of the carcasses at the time of necropsy was classified as 1 in only 14.3% (2/14) of cases, while the remaining 86% of cases were equally classified as 2 (28.6%; 4/14), 3 (28.6%; 4/14), and 4 (28.6%; 4/14). 

The NCC was classified as “good” in 28.6% (4/14) of cases, “moderate” in 28.6% (4/14) of cases, and “poor” in 21.4% (3/14) of cases; for the remaining cases (8, 9 and 10), the NCC was not determined because of their advanced decomposition status.

More detailed history, stranding data and diagnostic investigation results are described for each case in Table 3. Moreover, additional information on performed analysis and photos of almost all carcasses are reported in Supplementary Materials Table S1.

### 3.2. Diagnostic Investigations

#### 3.2.1. Histological and Immunohistochemical Investigations

Histological investigations were performed on 78.6% of cases (11/14); for cases 8 and 10, no samples were collected due to general tissue degradation, while for case 14, only samples from the gastrointestinal tract were collected.

Considering significant histopathological findings in the integumentary system, multiple lesions characterized by marked proliferation of the dermal papillae, multifocal hydropic degeneration, and keratinocytes hyperpigmentation at the level of the *stratum spinosum* referable to *Cetacean poxvirus* type 1 (CePV-1) were reported in the skin of case 7. Moreover, case 12 showed multiple skin lesions that tested positive for alphaHV and were identified as pyogranulomatous panniculitis, probably associated with a mixed infection.

**Figure 2:**
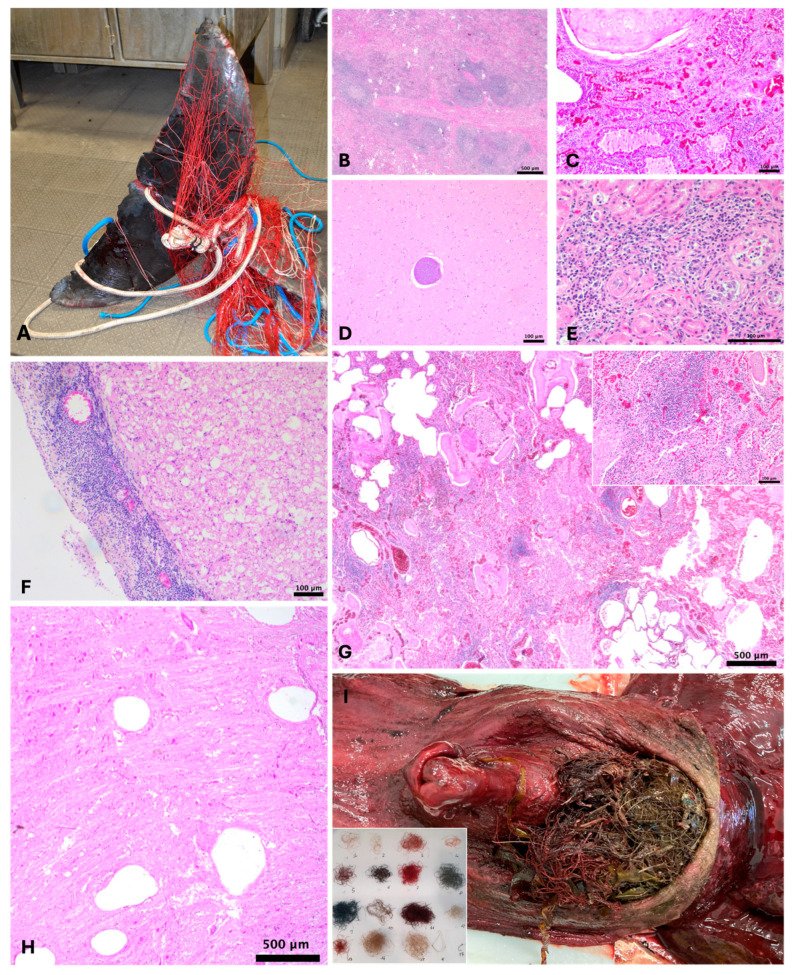
Macro and microscopic images of some of the animals selected for the study. (**A**) Case 7. Rope and nets around the tail. (**B**) Case 1. Marked and diffuse follicular lymphoid depletion in the mesenteric lymph node. Scale bar = 500 µm. H&E. (**C**) Case 1. Marked and diffuse lymphoplasmacytic and eosinophilic interstitial pneumonia associated with inflammatory edema and hyperemia in the lungs. Scale bar = 100 µm. H&E. (**D**) Case 5. *T. gondii* cyst at thalamus level in the CNS. Scale bar = 100 µm. H&E. (**E**) Case 4. Mild mononuclear interstitial nephritis associated with focal glomerular degeneration in the kidneys. Scale bar = 100 µm. H&E. (**F**) Case 5. Marked non-suppurative meningitis at cervical spinal level. Scale bar = 100 µm. H&E. (**G**) Case 4. Severe and diffuse granulomatous bronchopneumonia with multifocal area of alveolar emphysema in the lungs. Moderate hyperplasia of the bronchial musculature is also observed multifocally. Scale bar = 500 µm. H&E. Upper inset: magnification of the inflammatory infiltrate represented predominantly by macrophages and lymphocytes and associated with hyperemia of the pulmonary vessels. Scale bar = 100 µm. H&E. (**H**) Case 7. Severe gas embolism at pons level in the CNS. The tissue is replaced by clear spaces with no defined capsule, inflammation or gas forming bacteria. Scale bar = 500 µm. H&E. (**I**) Case 11. Marine litter (fishing line agglomerate) in the esophagus. Lower inset: 15 different types of fishing net and two hooks were found in the agglomerate.

In the lymphatic system, the main pathological findings were lymphoid depletion detected in the lymph nodes and/or in the spleen of cases 1, 2, 4, 5, 7, 12, and 13; this finding could be possibly associated with CeMV systemic infection in case 5 and with CeMV infection in the mesenteric lymph node of case 12. Additionally, case 1 reported marked lymphoid depletion in the mesenteric lymph node, possibly related to alphaHerpesvirus (alphaHV) infection (Figure 2B).

In the lungs, severe and diffuse granulomatous bronchopneumonia and multifocal eosinophilic granulomatous bronchopneumonia were detected in cases 4 and 5, respectively, possibly related to chronic parasitic infestation in both cases (Figure 2G). Regarding the cardiovascular system, severe gas embolism in subpleural and cerebral vessels was reported (Figure 2H).

In the digestive system, chronic eosinophilic cholangiohepatitis of parasitic origin associated with ductal fibrosis was found in the livers of cases 5 and 13. Also, focal pyogranulomatous gastritis of parasitic origin was reported in case 13.

Concerning the urinary system, case 4 showed mild multifocal chronic interstitial nephritis in association with local glomerular degeneration (Figure 2E). 

In conclusion, neuropathological examination revealed non-suppurative meningoencephalitis potentially associated with *T. gondii* systemic infection in case 1 and non-suppurative meningoencephalitis compatible with CeMV infection in the brain of case 12. Moreover, in case 5, severe non-suppurative meningoencephalitis and plexus choroiditis possibly related to CeMV systemic infection and *T. gondii* positivity in the CNS, was detected (Figure 2F). 

Immunohistochemistry investigations were performed for all cases except for 8, 10, 11, and 14. In detail, case 5 showed a positivity for *T. gondii* antigens in the CNS while positive results for Morbillivirus-specific antigens at the cerebral level were reported only for case 12.

#### 3.2.2. Bacteriological Investigations 

Bacteriological investigations were performed on 85.7% of cases (12/14); for cases 10 and 14, no samples were collected due to general tissue degradation.

In case 1, *L. grayi* was detected in the CNS without a reliable evaluation of the potential association with the non-suppurative meningoencephalitis; additionally, a systemic infection by *C. perfringens* and Pdd was also detected without a particular association with the reported findings. For case 2, systemic infection by Pdd (HlyAch positive) was detected. In case 4, *C. perfringens* was detected both in the lungs and kidneys. In case 7, *L. seeligeri* was detected in the CNS, but a reliable evaluation of the potential associated lesions could not be conducted. Moreover, a systemic infection by *Carnobacterium* spp. and *Serratia* spp. was reported in association with a subcutaneous and muscular abscessual lesion in the right lumbar paravertebral region. In case 9, *E. faecalis* systemic infection was reported in association with multifocal suppurative pneumonia. In case 11, systemic infection by *E. rhusiopathiae* was detected, although a reliable evaluation of the potential lesions associated could not be conducted because of tissue degradation. 

In case 13, both systemic infection by *C. perfringens* and Pdd were detected without any suggestive lesion.

#### 3.2.3. Biomolecular Investigations 

It was possible to perform biomolecular analyses on all cases selected for the study. In detail, *T. gondii* systemic infection was reported in cases 1 and 11, possibly associated with non-suppurative meningoencephalitis in case 1. Unfortunately, for case 11, it was not possible to perform histological investigations in the CNS due to severe autolysis of the tissue.

For case 5 and 6, *T. gondii* was detected in the brain and was possibly associated with non-suppurative meningoencephalitis in case 5, which also reported a systemic CeMV infection to which the same neuropathological findings could be related. 

For cases 6 and 8, CeMV was detected in the lungs and spleen, respectively.

For case 12, the detection of CeMV in the CNS and mesenteric lymph node was associated with non-suppurative meningoencephalitis and lymphoid depletion; additionally, specific Morbillivirus antigens were detected in the CNS by IHC.

Biomolecular evidence of *Herpesvirus* was reported at the systemic level in case 13, in the mesenteric lymph node of case 1, and in skin lesions of case 12. Considering lymphoid depletion as a pathological finding referable to HV, this finding was reported in both case 1 and case 13. 

Case 12 showed severe pyogranulomatous panniculitis, whereas panniculitis has been considered consistent with HV infection [69]. However, pyogranulomatous panniculitis in marine mammals has been more commonly associated with *Mycobacterium marinum* [70,71] but since this agent was not specifically examined in this study, the possibility of co-infection cannot be ruled out.

For all three cases (1, 12 and 13), molecular characterization identified the detected strain as alphaHV.

For case 7, CePV was detected in association with tattoo skin disease (TSD) and was subsequently sequenced as type 1.

#### 3.2.4. Serological Investigations

Serological investigations were performed only in 21.4% of cases (1, 2, and 3).

Antibodies for *T. gondii* were detected specifically in the serum, aqueous humor, and intracardiac clot of case 1 (1:40), which in fact reported a systemic infection by *T. gondii*. 

No evidence of *Morbillivirus* and *Brucella* spp. antibodies was reported in any of the analyzed cases.

### 3.3. Cause of Death Evaluation

Hypotheses on the cause of death were formulated for 64.3% of cases (1, 2, 3, 4, 5, 7, 9, 12 and 13). For the remaining 35.7% of cases (6, 8, 10, 11 and 14), the COD was undetermined due to the advanced DCC of the carcasses that did not allow us to define the likely COD with a reasonable margin of confidence.

In particular, COD was categorized as anthropic in 21.4% of cases (1, 4, and 7), specifically represented by fishery interaction in 100% of cases and as natural in 42.8% of cases (2, 3, 5, 9, 12, and 13), specifically represented by infectious diseases in 35.7% of cases (2, 5, 9, 12, and 13) and natal/perinatal disorders only in case 3.

Considering COD of anthropic origin, all 3 cases were related to fishery interaction, particularly categorized as by-catch with active fishing gear as a consequence of underlying pathologies.

In detail, case 1 showed evidence of confirmed fishery interaction due to the presence of rope and nets around the tail in association with good NCC and the presence of recent and abundant gastric content; case 4 reported net marks at the tail and thoracic level considered as signs of probable fishery interaction in association with good NCC and presence of recent and abundant gastric content; finally, case 7 was classified as confirmed fishery interaction due to disseminated gas bubbles in the main vessels (meningeal, mesenteric, pulmonary and coronary vessels, and renal capsule), the presence of recently ingested gastric content, moderate NCC, pulmonary, and vascular changes, disseminated congestion, and other unspecific lesions associated with underlying pathologies [72]. Considering gas bubbles, in the literature, this finding is reported also in animals stranded for other reasons, but when present in two or more organs, this finding resulted in significantly higher numbers in by-caught marine mammals [73].

COD of natural origin due to infectious diseases was related to bacterial infections in 14.3% of cases (2 and 9); in particular, case 2 reported systemic infections due both to *C. sordelli* and Pdd. For case 9, a systemic infection by *E. faecalis* in association with bilateral suppurative pneumonia was considered as the likely COD. 

COD related to viral infections was reported in 21.4% of cases (5, 12 and 13); in particular, case 5 was characterized by a severe non-suppurative meningoencephalitis and plexus choroiditis due to *T. gondii* and CeMV coinfection (viral—parasitic); case 12 showed non-suppurative meningoencephalitis due to CeMV infection (viral), and case 13 reported systemic coinfection due to alphaHV, Pdd, and *C. perfringens* (viral—bacterial).

In case 3, COD was instead related to natal/perinatal disorders due to congenital diencephalic damage (neuronal necrosis at thalamus level).

### 3.4. Toxicological Analysis 

HCB, PCBs, and DDTs were detected in all cases, and results for each animal are summarized in Table 4 along with their sex, extracted organic material (EOM%) and the percentage of immunosuppressive OCs (IS-OCs) on the total detected OCs.

EOM% ranged between 46.05% to 91.16% with a mean value of 74.58%. In all specimens the same pattern of relative abundance of the target contaminants was reported: PCBs > DDTs ≫ HCB. 

The highest HCB levels were detected in Case 2 (722.58 ng/g l.w.), while PCB and DDT levels were greatest in Case 11 (1,412,439.92 ng/g l.w.) and in Case 4 (168,936.08 ng/g l.w.), respectively. Regarding IS-OCs, it was observed that 13 out of the 14 animals had levels that accounted for more than 50% of the total OCs. Case 14 was the only one with the IS-OCs relative percentage below 50%, precisely 45.88%, and was also the one with the lowest levels of all the three OC groups. 

**Figure 3:**
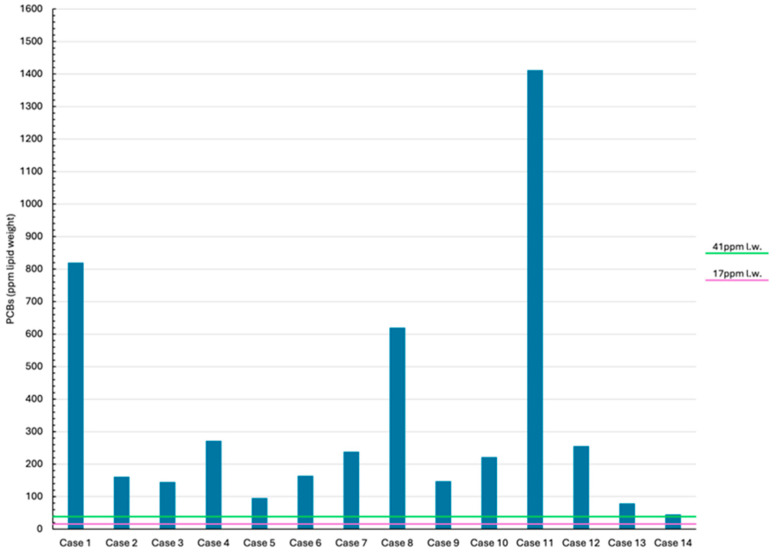
PCB levels expressed in ppm lipid weight (l.w.) were detected in the blubber samples of all study cases. A lower PCB toxicity threshold of 17 mg/kg l.w. was used for the onset of physiological endpoints in marine mammals; 41 mg/kg l.w. represents the highest PCB toxicity threshold published for marine mammals [74].

PCB levels recorded both in stranded bottlenose and striped dolphins are reported in Figure 3. The upper black dashed line (Figure 3) represents the PCB concentration threshold (PCBs = 41 mg/kg l.w.) for the highest PCB toxicity published for marine mammals based on marked reproductive impairment in ringed seals in the Baltic Sea [74]. The lower light red dashed line (Figure 3) represents the PCB threshold (PCBs = 17 mg/kg l.w.) which is known to cause adverse health effects and immunosuppression in marine mammals [75,76].

All cases exceeded both threshold levels of PCBs toxicity. Considering tissue concentrations of PCBs recorded in bottlenose dolphins (mean PCBs = 470,322.8 mg/kg l.w.) and striped dolphins (mean PCBs = 153,062.14 mg/kg l.w.), both were highly far beyond the PCB threshold values and, for bottlenose dolphins, the mean value was more than twice the average reported for striped dolphins.

**Figure 4:**
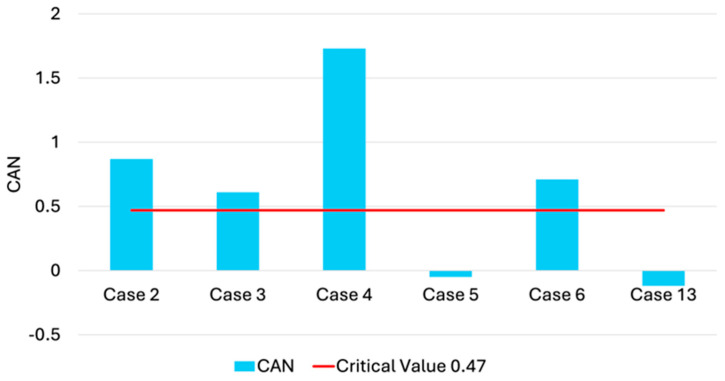
CAN values calculated for all the striped dolphins present in this study. The critical value of 0.47 is also indicated.

For the striped dolphins (cases 2, 3, 4, 5, 6, and 13) present in this study, the results of CAN [12] showed values exceeding the limit threshold of 0.47 in 66.7% of cases (2, 3, 4 and 6) which is an index of a high toxicological stress following exposure to DDT and PCBs (Figure 4).

### 3.5. GIT Analysis and Marine Litter Investigations

In order to evaluate the presence of macro-, meso- and microplastics in the GIT of stranded animals, a complete analysis of the GIT was performed in 43% of cases (5, 7, 11, 12, 13, and 14), as reported in Table 5 [52]; unfortunately, the remaining cases were not analyzed due to their advanced DCC and/or due to logistic problems in analyzing large volumes.

Gastric content was present in 50% of the analyzed cases; in detail, for cases 12 and 14, gastric content was reduced. The observance of macroscopic lesions in the stomach reported the presence of *P. gasterophilus* nodules in 83.3% of cases (5, 7, 12, 13, and 14). Analysis of marine debris in the gastrointestinal tract (GIT) of the organisms studied revealed a consistent presence of plastic debris, particularly meso- and microplastics, in all cases. Notably, no items larger than 25 mm (macroplastics) were observed in any of the samples. While plastic particles were generally found in both the stomach and the intestine, case 13 deviated from this pattern and showed no particles in the stomach.

Interestingly, mesoplastics were predominantly found in the intestinal tract, with a single case of their presence in the stomach of case 12. Microplastics, the smallest size category examined, were the most ubiquitous form of debris, widely distributed throughout the GIT of all analyzed cases. This ubiquitous presence of microplastics raises concerns about the potential ecological and health impacts.

## 4. Discussion

The Mediterranean Sea is a semi-enclosed marine ecosystem, which means that cetaceans inhabiting these waters are highly vulnerable to anthropic pressure ranging from intensive coastal development, fishing industry, marine traffic, and different types of pollution to climate change [7,77]. To evaluate the impacts of anthropic activities on cetaceans’ health, it is crucial to investigate routinely stranding events that are a fundamental source of information on cetacean population health status, allowing us to determine the causes of death and understand the main threats to which these populations are subjected to, including both anthropogenic and natural risks [7].

To the best of our knowledge, this is the first study that aims to assess the impact of anthropic activities on the health of cetacean populations specifically living in the Pelagos Sanctuary using a multidisciplinary approach.

In detail, out of 37 animals stranded along the Ligurian coastline between 2020 and 2022, it was possible to perform a complete *post mortem* examination on 62.2% (23/37) of cases. Among them, 61% (14/23) showed different evidence of anthropic interaction and were then selected for this study, regardless of whether or not there was a relationship with the cause of death.

One of the selection criteria to evaluate anthropic pressure on cetacean health in this study was the evidence of fishery interaction, since it is known that several Mediterranean cetaceans, especially coastal species such as common bottlenose dolphins, compete for prey of commercial interest that have been heavily exploited by human fisheries during the last decades [8]. Therefore, is particularly this spatial overlap that could promote interactions between cetaceans and fisheries [9,78].

Since 2020, with the application of Life DELFI’s framework for fishery interaction, it was possible to better assess cetacean *post mortem* findings and perform more precise diagnostic evaluations through a standardized and multi-tiered protocol [47]. According to Life DELFI’s annual report, by-catch represents the fishery interaction category recorded in the majority of cases in cetaceans stranded along the Italian coastline; in detail, evidence of fishery interaction was reported, respectively, in 20% and in 18.3% of all cetaceans stranded along the Italian coastline and subjected to a complete *post mortem* examination, during 2020 and 2021 [72].

Considering the study area, 30.4% (7/23) of stranded animals that went through a complete *post mortem* examination between 2020 and 2022 reported certain, probable, or suspected fishery interaction. Among the selected cases, 50% (7/14) of them showed certain, probable, or suspected fishery interaction, but only in 21.4% (3/14) of cases the likely cause of death was related to physical harm caused by fishery interaction, specifically by-catch with active fishing gear as a consequence of underlying pathologies.

It is important to emphasize the difference between an interaction with fishery causing or not causing the death of the animal; in fact, in the first case, the DCC can influence the hypothesis of the cause of death while the interaction can always be ranked as certain, probable, or suspected [47].

In fact, all 3 cases (1, 4, and 7) with a COD of anthropic origin had a DCC of 2, a good NCC and the presence of recently ingested gastric content at the time of death; the interaction with fishery was then considered the ultimate cause of death, although underlying pathologies of multifactorial etiology (bacterial, viral, and protozoan) could have predisposed the animals to the mentioned interaction [14,72].

Considering the remaining 4 cases (8, 9, 10, and 11) of fishery interaction selected for this study, half of them had a DCC of 3 and the other half a DCC of 4; the COD remained undetermined in 75% of cases (3/4) and was classified as natural in one case. The interaction was rated as suspected in 75% of cases (3/4), particularly categorized as by-catch with undetermined fishing gear, while for 25% of cases (1/4), the interaction was rated as certain and categorized as ingestion [14,72]. Overall, 86% (6/7) of fishery interaction reported in this study was categorized as by-catch, which is in fact particularly reported in the Tyrrhenian marine region [72].

The species primarily involved in fishery interaction in this study was bottlenose dolphins (86%; 6/7) and the majority (57.1%; 4/7) of them were adults males (71.4%; 5/7). As reported by Life DELFI’s annual report, adult male common bottlenose dolphins represent the most involved species in fishery interactions, confirming the behavioral habits of this Mediterranean coastal species [72].

Moreover, beyond considering direct damages due to fishery interaction, we must consider the reduction in prey availability that affects cetacean food resources, implying higher energetic costs for dolphins to obtain their daily food intake, reducing the adaptability of these species to other environmental changes (e.g., climate change, pollution, infectious diseases, etc.) [8]. In this context, inadequate nutrition in Mediterranean striped dolphins may have contributed to an epizootic outbreak [79] and could be responsible for the significantly elevated age at sexual maturation observed in this area compared to other conspecific populations in regions with more abundant food resources [80,81].

Another selected criterion to evaluate anthropic pressure on cetacean health in this study was the detection of terrestrial pathogen-associated disease in stranded animals.

We must consider that infections by specific pathogens have likely occurred for thousands of years, maintaining an equilibrium between populations and pathogens, as seen in other species [82]. However, environmental changes caused by human activities are believed to have disrupted this balance by weakening population immune responses, increasing stress, and facilitating the introduction of new pathogens, among other effects [83]. Biological pollution is an emerging issue, with the findings of terrestrial pathogens in marine mammals, especially in inshore species such as common bottlenose dolphin, which incur higher risks than pelagic cetaceans due to habitats often strongly altered by anthropogenic factors [83]. Considering the Ligurian area, several cases of terrestrial pathogen-associated diseases have been reported in the last decade [22,25,28,29,32,34,36,84]. In our study, 50% (7/14) of total selected cases reported terrestrial pathogen-associated disease or systemic infections, regardless of whether or not there was a relationship with the cause of death.

In detail, *T. gondii,* a zoonotic coccidian protozoan considered as a primary pathogen for cetaceans and responsible for toxoplasmosis [22], was detected in 21.4% (3/14) of cases, causing systemic infections in two bottlenose dolphins (case 1 and 11) and a non-suppurative meningoencephalitis with positive IHC in the brain of one striped dolphin (case 5).

Despite the limited number of cases, the systemic infection by *T. gondii* detected through biomolecular investigations in two bottlenose dolphins without the occurrence of specific inflammatory lesions, when compared to the pathological findings observed in the brain-positive striped dolphin, seems to agree with the marked susceptibility of striped dolphin to the infection [22,25,32]. The severe disease patterns described for this species could be related to the absence of mutual host–parasite coevolution, which is present in coastal species like the bottlenose dolphin. These dolphins experience more frequent exposure to *T. gondii* without developing the disease [14,32].

The number and the nature of infections show how *T. gondii* has spread in coastline waters likely affected by anthropic pressure, along with the prolonged resistance of protozoan oocysts even in sea water [7]. Considering offshore species like the striped dolphin, global maritime trade is thought to be the origin of the dissemination of this agent, due to ship run-off waters with poor hygienic conditions and the presence of rodents, cats, or contaminated soil onboard [83].

Considering another terrestrial and zoonotic pathogen, in addition to *T. gondii* systemic infection mentioned above for one bottlenose dolphin (case 11), a systemic infection by *E. rhusiopathiae* was also detected for this case.

This ubiquitous Gram-positive bacterium can persist for extended periods in the environment, including marine locations [85]. Necropsy findings in case of *E. rhusiopathiae* septicemia vary and may include pulmonary edema, vascular congestion, multiple hemorrhages, dermal infarctions resulting in rhomboidal plaques or ulcerative lesions, enlarged and edematous lymph nodes, and bacterial emboli [86,87,88]; however, in our case, gross lesions were subtle or non-specific; by performing routine microbiology analysis, it was possible to identify *E. rhusiopathiae* in multiple tissues. During *post mortem* examination, there were not the characteristic skin lesions, and the advanced autolysis of internal organs did not allow us to collect significant pathological findings.

The dermatologic and acute septicemic forms have been reported in several cetacean species, including captive and free-ranging individuals, representing the most susceptible marine mammals to this disease [86]. Compared to captive animals, free-ranging individuals may die during the acute phase, and the fact that subacute or chronic forms are rarely reported may indicate that these animals are able to overcome the disease [86,88] unless a strong immune suppression is affecting the animal [87].

The possible exposure of humans to costal species such as bottlenose dolphins presents potential risks of zoonotic infection by infectious agents that may be carried by cetaceans both as components of their normal flora or as pathogens; the likely transmission from dolphins to humans may be caused by a common use of coastal waters [89].

Other relevant terrestrial pathogens reported in this study belong to the bacterial genus *Clostridium*; in detail, *C. perfringens* systemic infection was detected in 14.3% (2/14) of cases (case 1 and 13), while a systemic infection by *C. sordellii* was reported in one case (case 2). *Clostridium* spores generally are able to survive in water for a long time [90], and this could be a source of infection for marine animals and humans.

In particular, *C. perfringens* is a Gram-positive bacterium and is a common organism in the environment as well as part of human and animal intestinal microbiota, but it is also known as an opportunistic pathogen that can cause serious infection in dolphins via skin wounds [91]. *C. perfringens* has been reported in the literature as a cause of death and as an etiology of gas bubble accumulation in cetaceans [92,93,94]. Considering our cases (1 and 13), no specific gross and histological lesions were reported, but, in both cases, a systemic infection by Pdd was also detected in association with a systemic infection by *T. gondii* in case 1 and a systemic infection by alphaHV in case 13.

In this regard, since histopathological alterations in co-infections can be a consequence of the overlapping of two or more pathogens, the specific pathogenesis of lesions cannot always be ruled out [53].

Considering *C. sordellii,* this agent can be generally detected in soil and the gastrointestinal tract of animals; occasionally, virulent strains are able to produce toxins [77]. In the literature, *C. sordellii* has been described as cofactor in septicemic infections in cetaceans [95] and as COD in one Atlantic white-sided dolphin (*Lagenorhynchus acutus*) stranded in the North Sea which died due to *C. sordellii* septicemia [96]. For our case (case 2), specific pathological findings were not reported, partly because of autolyzed tissues at the time of necropsy; however, even in this case, a systemic infection by Pdd was also detected.

Pdd, which is not a bacterium of terrestrial origin, is transmitted through water in the presence of continuous solutions at the skin level [97,98] and is considered a primary pathogen for a wide range of aquatic species. In cetaceans, it has been isolated both from healthy and stranded dolphins and is commonly considered an opportunistic agent, in the absence, however, of a clear interpretation of the pathogenic role played [23]. Coinfection due to Pdd and other pathogens has been previously described in stranded cetaceans [23,98], with CeMV acting as a predisposing factor to the infection due to induced immunosuppression [99].

Of interest for this study was the isolation of two other bacteria that caused a systemic infection in a stranded bottlenose dolphin (case 7): *Serratia* spp. and *Carnobacterium* spp. In detail, *Serratia* spp., an opportunistic pathogen normally found in the environment and causing infections in immunocompromised human patients [100], is not widely reported in cetaceans so far, except as *S. marcescens* [101,102]. Regarding *Carnobacterium* spp., this bacterium has been largely isolated from the skin of healthy bowhead whales (*Balaena mysticetus*) [103] and is commonly found in aquatic environments as part of the microbiome of some teleosts and sharks, but in the case of stress-induced immunosuppression, these bacteria can become pathogenic and cause systemic infection [103,104].

This may have been the case for the bottlenose dolphin in our study, where a subcutaneous and muscular abscess in the right lumbar paravertebral region could have been the entryway for these opportunistic pathogens, associated with a multicentric reactive lymphadenopathy.

Another relevant pathogen reported in this study is *E. faecalis*, which caused a systemic infection in a stranded bottlenose dolphin (case 9), in association with a multifocal suppurative pneumonia and a reactive lymphadenopathy. *E. faecalis* is a Gram-positive bacterium and is the major commensal bacteria in the GIT of humans and other mammals; it has also been reported as opportunistic pathogen both in humans and domestic animals, causing several pathological findings such as orchitis, septicemia, mastitis, and endocarditis, and a pyogranulomatous dermatitis in a captive spotted seal (*Phoca larga*) [105].

Finally, two other pathogens of terrestrial origin isolated from the brain of two bottlenose dolphins were *L. greyi* and *L. seeligeri*, detected, respectively, in the brains of cases 1 and 7, without specific associated lesions.

In conclusion, these pathogens are not considered as native to the marine environment but can possibly reach seawater via run-off freshwaters feeding rivers during unusual rainy periods and severe flooding events, especially in the Ligurian area [25,106]. Although these are usually single-case reports, these findings could highlight the risk of biological marine pollution from different mainland activities as a relevant threat to marine mammal conservation [36].

As highlighted in our study, despite the small sample size of animals, the frequent presence of coinfections and opportunistic pathogens infections in stranded dolphins would support the hypothesis that lowering the population immune response through chemical pollution, depressing food supplies, increasing stress, and facilitating the introduction of alien infectious agents has increased the emergence and severity of several diseases in pinnipeds and cetaceans [83]. In these cases, opportunistic pathogens may take over, increasing the level of debilitation of the animal with consequent death and/or stranding [4,83,107].

Although the use of these persistent organic pollutants (POPs) is now banned, significant levels of these substances still persist in the environment, affecting cetacean populations worldwide [7]. Marine mammals are particularly vulnerable to the high bioaccumulation of these pollutants due to their high trophic level, long life spans, and limited ability to metabolize and excrete these compounds [14,74].

While the direct effects of POPs are difficult to assess, numerous studies have documented their toxic effects including immune suppression, endocrine disruption, reproductive impairment, and carcinogenic effects in various mammalian species, including marine mammals [7,74].

For this reason, the levels of OCs such as HCB, PCBs, and DDTs, known for their toxic effects such as immunosuppression and reproductive impairment in marine mammals [74], were evaluated in each case of this study, to assess potential toxicological hazard [12,48].

In this study, toxicological investigations of DDT, PCBs, and HCB in the blubber of examined animals consistently showed the pattern PCBs > DDTs ≫ HCB. This pattern aligns with the accumulation trend reported in cetaceans living in the Mediterranean Sea in recent years, with PCBs consistently showing the highest levels, especially in the northwestern part of the basin [2,108,109].

It is important to note that in all cases, the PCB levels exceeded the 17 mg/kg l.w. threshold for PCB-induced adverse health effects [75]. Even when using the highest reported PCB toxicity threshold for marine mammals of 41 mg/kg l.w. [74], all of the stranded dolphins in this study exceeded this level. Furthermore, all cases greatly exceeded the PCB threshold values, with the mean value for bottlenose dolphins being more than double that of striped dolphins.

These threshold values are not absolute, but should be used as a guide for determining whether PCB exposure levels are likely to cause significant immunotoxic effects based on empirical data [14].

For the striped dolphins in this study, the CAN value was also calculated to assess toxicological stress in addition to PCB levels. The CAN values exceeded the threshold of 0.47, an indicator of high toxicological stress from exposure to DDT and PCBs, in 66.7% of cases [12]. In the two cases (5 and 13) that did not exceed the 0.47 threshold, the CAN calculation showed that DDTs + PCBs levels were not considered toxicologically dangerous for Mediterranean striped dolphins, although PCB levels still exceeded the 17 mg/kg l.w. threshold for harmful effects, including immunosuppression.

Lastly, it is noteworthy, though not uncommon, that one newborn striped dolphin (case 3), which died from neonatal or perinatal pathologies, had a CAN level (0.61) that exceeded the threshold. This is consistent with known phenomena where females detoxify during gestation and even more during lactation, transferring much of their contaminant load to their offspring through milk [110].

In conclusion, regarding marine litter pollution, due to sampling limitations and the need to process large sample volumes, the study of microplastic ingestion by cetaceans presents unique methodological challenges. However, the implementation of a multidisciplinary approach using a novel multi-sieve tool in this study facilitated the successful isolation of even the smallest particles within distinct sections of the gastrointestinal tract. While the absence of macroplastics in our findings may be due to differences in feeding ecology and prey preferences compared to other studies [19], the consistent presence of microplastics is of great concern given their widespread distribution in the Pelagos Sanctuary [21].

This highlights the need for further research into the long-term impacts of microplastics, which are known to have detrimental effects on marine organisms, including physical harm, inflammation, and the potential to vector other pollutants. Moreover, since there is increasing evidence that plastic waste can contribute to environmental persistence of bacteria released through human and animal discharges, both macro and micro-litter may promote the dissemination of zoonotic pathogens in the marine environment [111].

To fully understand the ecological and toxicological impacts of marine litter pollution and to develop effective mitigation strategies, continued research and monitoring efforts such as those undertaken in this study are essential.

## 5. Conclusions

The causes of strandings are generally multifactorial and it is important to develop an overall view, since it is often the synergistic action of multiple factors that has a negative impact on cetacean health, up to the death of the animal itself.

Considering the results of this study, we can confirm high levels of anthropic pressure on cetaceans living in the Ligurian area of the Pelagos Sanctuary, with relevant findings concerning fishery interaction, OCs pollution, and terrestrial pathogen-associated diseases.

These data highlight the need for ongoing surveillance and monitoring studies on stranded cetaceans, employing a multidisciplinary and standardized approach to enhance our understanding of the impact of human activities. Conservation policy concerning cetacean populations should incorporate stranding data as a crucial contribution in monitoring cetacean’s health and their habitat under a One Health perspective.

## Figures and Tables

**Table 1:** Summary of the diagnostic investigations conducted for each case, listed in chronological order.

Case ID	IZS Code	Histology	Immunohistochemistry (IHC)	Bacteriology	Biomolecular (PCR)	Serological
1	51352/20	x	x	x	x	x
2	60669/20	x	x	x	x	x
3	80029/20	x	x	x	x	x
4	87243/20	x	x	x	x	
5	26362/21	x	x	x	x	
6	30434/21	x	x	x	x	
7	41716/21	x	x	x	x	
8	50946/21			x	x	
9	53638/21	x	x	x	x	
10	61838/21				x	
11	73951/21	x		x	x	
12	177/21	x	x	x	x	
13	8319/22	x	x	x	x	
14	63831/22	x ^1^			x	

^1^ = limited samples.

**Table 2:** Results and criteria for which each case was selected for this study are reported in the following table. Each case has been selected on the basis of the presence of one or more factors of anthropic interaction, including fishery interaction, toxicological stress, and terrestrial pathogen-associated lesions. Considering the presence of marine litter in GIT, this parameter was not considered as a selection criterion but was nevertheless considered retrospectively for the analyzed cases.

Case ID	Fishery Interaction [47]	Toxicological Stress [12,14]	Terrestrial Pathogens- Associated Lesions	Marine Litter in the GIT [52]
1	x	x	x	NE
2		x	x	NE
3		x		NE
4	x	x		NE
5		x	x	x
6		x		NE
7	x	x	x	x
8	x	x		NE
9	x	x	x	NE
10	x	x		NE
11	x	x	x	x
12		x		x
13		x	x	x
14		x		x

Legend: NE = not evaluated.

**Table 3:** Cetaceans stranded along the Ligurian coastline between 2020 and 2022 were selected for the study. For each individual detailed information are reported: date and location of stranding, species, sex, total body length (TBL) (cm) and weight (kg), estimated age class, decomposition condition category (DCC), nutritional condition category (NCC), gastric contents, main macro and microscopic findings, pathogens and helminths detected, classification of the cause of death (COD), origin, and sub-category.

Case ID	Stranding Date	Stranding Location	Species	Sex	TBL (cm); Weight (kg)	Estimated Age Class	DCC	NCC	Gastric Content	Main Macro- and Microscopic Findings	Pathogens and Helminths Detected	COD	Origin	Sub-Category
**1**	13/07/2020	San Fruttuoso (GE)	Bottlenose dolphin	M	310 cm	Adult	2	G	Present	**Macro:** nets and ropes around the caudal peduncle; gas embolism in the renal capsule and mesenteric vessels; severe hyperplasia of MES. and PUL. lymph.; pulmonary oedema. **Micro:** alveolar oedema and marked mononuclear-eosinophilic interstitial pneumonia; generalized eosinophilic lymphadenitis and lymphoid depletion in MES. and PUL. lymph.; moderate lymphoplasmacytic enteritis; NS meningoencephalitis; muscular hyaline degeneration with wavy cells and atrophy of myocytes.	***T. gondii*** (systemic infection); ***T. gondii*** antibodies (1:40) (serum, AH and intracardiac clot); **α-Herpesvirus** (alphaHV) (MES. lymph.); **Pdd** (systemic infection); ***Listeria grayi*** (CNS); ***Penicillium*** spp. (lungs, lymph nodes); ***Clostridium perfringens*** (systemic infection)	Anthropic	Fishery interaction	By-catch consequence of underlying pathologies
**2**	23/08/2020	Noli (SV)	Striped dolphin	M	126 cm; 27 kg	Juvenile	3	M	Scarce	**Macro:** Gelatinous oedema of the blubber in ventral abdominal region; mild infestation by *Phyllobotrium delphini* in the blubber of peri genital region; hemorrhagic CSF and peri-medullary tissue congestion in the cervical region. **Micro:** multifocal alveolar emphysema associated with mononuclear and eosinophilic multifocal interstitial pneumonia; lymphoid depletion with sinus histiocytosis in PS. lymph.; pyogranulomatous tonsillitis associated to lymphoid hyperplasia.	**Pdd** (systemic infection; HlyAch positive); ***P. delphini*** (blubber); ***Clostridium sordelli*** (systemic infection)	Natural	Infectious	Bacterial
**3**	01/11/2020	Albisola Superiore (SV)	Striped dolphin	M	90 cm; 9.5 kg	Newborn	1 **	G	Scarce	**Macro:** focal hemorrhagic lesions on the ventral margin of the right lobe of the liver (compatible with euthanasia). **Micro:** neuronal necrosis at thalamus level.		Natural	Neonatal/perinatal pathologies	
**4**	23/11/2020	Spotorno (SV)	Striped dolphin	M	200 cm; 88 kg	Adult	2	G	Present	**Macro:** net marks at tail and thoracic level; peritoneal infestation by multiple parasite cysts; infestation by *Phyllobotrium delphini* in the blubber; *Anisakis* spp. in the stomach associate to multifocal gastritis; mesenteric and coronary vessel gas embolism (score: III/IV); sub meningeal vessel gas embolism (score: IV/IV) [51]; multiple *Campula* spp. parasites in liver and pancreas; severe nematodes broncho-pulmonary infestation associated with multifocal emphysema and congestion in the lungs; hemorrhagic CSF. **Micro:** moderate generalized follicular lymphoid depletion and hypercellularity of the sinuses in PS., TB. and PUL. lymph.; lymphoid depletion in the spleen; severe and diffuse granulomatous bronchopneumonia and hyperplasia of the bronchial musculature; mild chronic interstitial nephritis.	***Clostridium perfringens*** (lungs and kidney); **Pdd** (lungs); ***P. delphini*** (blubber); ***Monorygma grimaldii*** (peritoneum); pulmonary nematodes (*Brucella* spp +); ***Pholeter gastrophilus*** (stomach)	Anthropic	Fishery interaction	By-catch consequence of underlying pathologies
**5**	17/03/2021	Ventimiglia (IM)	Striped dolphin	M	178 cm; 50 kg	Juvenile	3	P	Absent	**Macro:** subcutaneous infestation by larval cestodes (*P. delphini*); gelatinous oedema of the blubber; multiple *Campula* spp. parasites in liver and pancreas; moderate lymphoid hyperplasia in PS. lymph.; moderate meningeal hyperemia. **Micro:** moderate chronic cholangiohepatitis; diffuse lymphoplasmacytic and eosinophilic enteritis with marked hyperplasia of associated lymphoid tissue; moderate generalized lymphoid depletion and hypercellularity of the sinuses; moderate lymphoplasmacytic enteritis; moderate and multifocal eosinophilic granulomatous bronchopneumonia and multifocal alveolar emphysema; severe NS meningoencephalitis and plexus choroiditis.	***P. gastrophilus*** (stomach); ***P. delphini*** (blubber); ***T. gondii*** (CNS; IHC +); **CeMV** (systemic infection)	Natural	Infectious	Coinfection (viral— parasitic)
**6**	29/03/2021	Arenzano (GE)	Striped dolphin	ND	120 cm *; 40 kg	ND	4	P	ND	**Macro:** complete evisceration of the abdominal cavity and ablation of the distal part of the carcass (*post mortem* predation); gelatinous oedema of the blubber.	**CeMV** (lungs); ***T. gondii*** (CNS); **Pdd** (systemic infection, HlyAch positive)	ND	ND	ND
**7**	25/04/2021	San Fruttuoso (GE)	Bottlenose dolphin	M	241 cm; 173.5 kg	Adult	2	M	Present	**Macro:** subcutaneous infestation by larval cestodes (*P. delphini*); subcutaneous-muscular abscess in the right lumbar paravertebral region; multifocal pulmonary parasitic granulomas; granulomatous gastritis; severe gas embolism in CNS, meningeal, mesenteric, and coronary vessels, renal capsule and lung (score: IV/IV) [51]; hemorrhagic CSF. **Micro:** marked proliferation of the dermal papillae, multifocal hydropic degeneration and hyperpigmentation of the keratinocytes at the level of the stratum spinosum; multifocal alveolar emphysema and edema; marked hepatic congestion; multifocal pyogranulomatous enteritis; moderate lymphoid depletion in PS., MES. and TB. lymph.; severe gas embolism in subpleural and cerebral vessels.	***Carnobacterium*** spp. (systemic infection); ***Serratia*** spp. (systemic infection); ***Listeria seeligeri*** (CNS); ***Cetacean poxvirus 1*** (CePV) (skin lesion); ***P. gastrophilus*** (stomach); ***P. delphini*** (blubber)	Anthropic	Fishery interaction	By-catch consequence of underlying pathologies
**8**	06/06/2021	Margonara (SV)	Bottlenose dolphin	F	270 cm	Adult	4	ND	Absent	**Macro:** amputation of dorsal and caudal fins; fishing line around the thorax associated with a linear skin lesion.	***T. gondii*** (spleen, heart); **CeMV** (spleen)	ND	ND	ND
**9**	12/06/2021	Savona	Bottlenose dolphin	M	124 cm; 30 kg	Newborn/calf	3	ND	Scarce	**Macro:** amputation of the caudal fin; generalized blubber gelatinous edema; multifocal bilateral suppurative pulmonary nodules.	***Enterococcus faecalis*** (systemic infection); ***T. gondii*** (liver)	Natural	Infectious	Bacterial
**10**	09/07/2021	Sestri Levante (GE)	Bottlenose dolphin	M	153 cm *; 31 kg	Juvenile	4	ND	Absent	**Macro:** amputation of the caudal fin; circular cut injury of the peduncle.		ND	ND	ND
**11**	10/09/2021	Andora (SV)	Bottlenose dolphin	F	190 cm; 130 kg	Juvenile	3	M	Absent	**Macro:** multifocal parasitic skin lesions (*Pennella* spp.); subcutaneous infestation by larval cestodes (*P. delphini*); foreign body in the esophagus referable to marine litter (fishing line agglomerate).	***T. gondii*** (systemic infection); **CeMV** (lung, urinary bladder); ***Erysipelothrix rhusiopathiae*** (systemic infection); **Pdd** (lung, PS. lymph.); ***Pennella*** spp. (skin); ***P. delphini*** (blubber)	ND	ND	ND
**12**	24/12/2021	Savona	Bottlenose dolphin	F	280 cm; 211 kg	Adult	2	G	Scarce	**Macro:** muscular and subcutaneous hematoma in the left prescapular region; moderate pulmonary oedema associated with foam in trachea; **Micro:** multifocal pyogranulomatous panniculitis; pyogranulomatous tonsillitis; granulomatous gastritis; lymphoplasmacytic endometritis; lymphoid depletion in spleen and MES. lymph.; NS meningoencephalitis.	**CeMV** (CNS and MES. lymph.; IHC + CNS); **α-HV** (skin lesions); ***P. gastrophilus*** (stomach)	Natural	Infectious	Viral
**13**	30/01/2022	Imperia	Striped dolphin	M	190 cm; 86.5 kg	Juvenile	1	M	Absent	**Macro:** severe subcutaneous and muscular infestation by larval cestodes *(P. delphini*); multifocal parasitic cysts in the peritoneum (*M. grimaldii)*; severe meningeal hyperemia. **Micro:** severe multifocal chronic cholangiohepatitis; focal pyogranulomatous gastritis; moderate lymphoid depletion with sinus histiocytosis in PS. lymph.; lymphoid depletion in spleen; mononuclear and eosinophilic multifocal interstitial pneumonia; severe pyogranulomatous tonsillitis; oedema in the white matter of the frontal cortex.	***M. grimaldii*** (peritoneum); ***P. delphini*** (blubber); ***P. gastrophilus*** (stomach); **α-HV** (systemic infection); **Pdd** (systemic infection); ***Clostridium perfringens*** (systemic infection)	Natural	Infectious	Coinfection (Viral— bacterial)
**14**	02/08/2022	Bocche di Magra (SP)	Bottlenose dolphin	M	163 cm; 45 kg	Juvenile	4	P	Scarce	**Macro:** generalized severe autolysis.	**Pdd** (sytemic infection)	ND	ND	ND

Legend: M = male; F = female; ND = not determined; DCC = decomposition condition category; NCC = nutritional condition category (G, good; M, moderate; P, poor); CNS = central nervous system; AH = aqueous humor; lymph. PS. = prescapular lymph node; lymph. MES. = mesenteric lymph node; lymph. PUL. = pulmonary lymph node; lymph. TB. = tracheobronchial lymph node. HlyAch = samples screened by PCR for hemolysins genes (*dly*, *hlyA_pl_*, *hlyA_ch_*). * For these cases the length was only estimated due to missing parts. ** Case stranded alive and subsequently euthanized.

**Table 4:** Levels of HCB, DDTs, PCBs, and IS-OCs in the blubber of different specimens of *Tursiops truncatus* and *Stenella coeruleoalba* (ID) expressed in ng/g lipid weight (l.w.). For IS-OCs the respective percentages of total OCs are reported. EOM% = Extracted Organic Material Percentage.

ID	Species	Sex	EOM%	HCB	PCBs	DDTs	IS-OCs
Case 1	*Tursiops truncatus*	M	70.21	156.59	820,327.62	104,406.43	388,104.79 (59.77%)
Case 2	*Stenella coeruleoalba*	M	91.16	722.58	161,280.92	97,662.73	160,362.31 (67.75%)
Case 3	*Stenella coeruleoalba*	M	86.24	643.62	145,369.74	88,380.32	140,038.41 (69.28%)
Case 4	*Stenella coeruleoalba*	M	79.48	292.75	271,817.39	168,936.08	257,341.30 (73.41%)
Case 5	*Stenella coeruleoalba*	M	77.57	208.13	96,221.77	45,858.19	72,472.58 (65.66%)
Case 6	*Stenella coeruleoalba*	ND	82.51	151.82	164,384.14	94,781.52	148,029.00 (69.18%)
Case 7	*Tursiops truncatus*	M	86.57	240.59	238,671.54	30,885.39	134,473.91 (57.57%)
Case 8	*Tursiops truncatus*	F	46.05	239.81	620,120.65	71,889.05	178,991.43 (56.15%)
Case 9	*Tursiops truncatus*	M	82.46	372.01	147,827.16	23,447.89	83,712.30 (59.14%)
Case 10	*Tursiops truncatus*	M	73.31	443.92	222,053.22	30,386.90	105,309.60 (56.80%)
Case 11	*Tursiops truncatus*	F	49.71	394.96	1,412,439.92	167,724.84	459,438.61 (58.48%)
Case 12	*Tursiops truncatus*	M	50.25	156.68	255,656.98	34,007.92	85,935.56 (59.01%)
Case 13	*Stenella coeruleoalba*	F	88.90	129.74	79,298.93	33,677.85	65,134.81 (64.78%)
Case 14	*Tursiops truncatus*	M	79.77	27.42	45,485.26	3679.57	18,004.87 (45.88%)

**Table 5:** The presence of gastric content and macroscopic lesions are reported below. The presence of marine litter both in the stomach and intestine is divided according to the size of particles into macro-, meso- and microlitter.

Case ID	Presence of Gastric Content	Macroscopic Gastric Lesions	Marine Litter in the Stomach			Marine Liter in the Intestine		
			Macro	Meso	Micro	Macro	Meso	Micro
5		3 gastric ulcers in the I concameration + *P. gastrophilus* nodules in the II and III concameration			x			x
7	x	*P. gastrophilus* nodules in the II concameration			x		x	x
11							x	x
12	x	*P. gastrophilus* nodules in the II concameration		x	x		x	x
13		Multiple *P. gastrophilus* nodules in the I– II concameration					x	x
14	x	*P. gastrophilus* nodules in the III concameration			x		x	x

## Data Availability

The data presented in this study are available within the article and the Supplementary Materials.

## Supplementary Materials

The following supporting information can be downloaded at: https://www.mdpi.com/article/10.3390/ani14223207/s1, Table S1: Detailed data on cetaceans stranded along the Ligurian coastline between 2020 and 2022. Figure S1: Photos of the carcasses of *T. truncatus* and *S. coeruleoalba* stranded along the Ligurian coastlines between 2020 and 2022.
